# Antioxidant, Antimicrobial, Cytotoxic, and Protein Kinase Inhibition Potential in* Aloe vera* L.

**DOI:** 10.1155/2019/6478187

**Published:** 2019-07-28

**Authors:** Huma Tariq, Muhammad Zia, Ihsan-ul-Haq, Syed Aun Muhammad, Shujaat Ali Khan, Nighat Fatima, Abdul Mannan, Arshad Mehmood Abbasi, Mingxing Zhang

**Affiliations:** ^1^Department of Pharmacy, COMSATS University Islamabad (Abbottabad Campus), Pakistan; ^2^Department of Biotechnology, Quaid-i-Azam University, Islamabad 45320, Pakistan; ^3^Department of Pharmacy, Quaid-i-Azam University, Islamabad 45320, Pakistan; ^4^Institute of Molecular Biology and Biotechnology, Bahauddin Zakariya University, Multan, Pakistan; ^5^Department of Environment Sciences, COMSATS University Islamabad (Abbottabad Campus), Pakistan; ^6^The First Affiliated Hospital/School of Clinical Medicines, Guandong Pharmaceutical University, Guangzhou 510006, China

## Abstract

*Aloe vera* is a multifunctional plant that has gained acceptance as an excellent home remedy source in Asia and the world. The present study was intended to evaluate the phytochemical contents and* in vitro* antioxidant, antimicrobial, antileishmanial, and protein kinase inhibition activities in different fractions of* A. vera* leaf. Methanolic extract of* A. vera *leaves was fractionated using column chromatography and ten fractions (AV1-AV10) were obtained. Phenolics composition, antioxidant, antimicrobial, antileishmanial, and protein kinase inhibition activities were evaluated using standard protocols. Well-known compounds of* A. vera* were used for in silico study against enzymes involved in brine shrimp and antileishmanial and hyphae formation inhibition assay on the basis of results. Five fractions (AV3 to AV7) possess potential total phenolics and flavonoids contents along with significant biological activities. AV4 fraction exhibited the highest total phenolics content 332.4 ± 32.6*μ*g GAE/mg and total antioxidant activity 150.4 ± 25.815*μ*g AAE/mg determined by phosphomolybdenum complex assay. Fraction AV6 showed 95% antileishmanial effect as well as the lowest LD_50_ value of 0.5305*μ*g/mL in brine shrimp lethality assay. The Protein Kinase inhibition potential in* A. vera* leaves was determined for the first time and three fractions AV1, AV6, and AV7 depicted activity with the highest zone of inhibition up to 21±0.5mm (AV7). Docking analysis showed that* A. vera* contains anthraquinones, anthrones, chromones, and polysaccharides responsible for synergistic cytotoxic, antileishmanial, antibacterial, and antioxidant potential of this plant. Therefore, with more studies,* A. vera* could probably have the potential to be used for drug development against leishmaniasis.

## 1. Introduction

The history of development of medicinal components is based on the fact that over the centuries the natural products such as taxol, artemisinin, and morphine were used to cure a number of diseases [1]. The natural products are more acceptable than purely synthetic products because they match biological intermediates and endogenous substances and have suitability in active transport mechanisms [2]. Nowadays, plants based secondary metabolites extracted from crude extract or fractions are good source of diverse chemical structures that show potent biological profile and pharmacological activities [3, 4].

Thousands of polyphenolics compounds such as phenolics and flavonoids have been discovered so far. These compounds retain different antioxidant, antibacterial, anticancerous, antiviral, and anti-inflammatory activities and antiatherosclerotic properties [5, 6]. The pharmacological and physiological potentials of phenolic compounds depend on their free radical scavenging and antioxidant activities and properties to maintain the activity of enzymes responsible for detoxification [7]. A very unique health effect of flavonoids is that they are cardioprotective due to inhibition of LDL oxidation [8, 9]. The ability of flavonoids to transfer stable free radicals and metal catalyst chelation provides protection to the biological systems [10], antioxidant enzymes activation [11], alpha-tocopherol radical reduction [12], and inhibition of enzyme oxidases [13].

Globally, infectious diseases are considered major cause of mortality and morbidity each year. The infectious disorders are caused by organisms such as bacteria, viruses, fungi, or parasites [14, 15]. Among bacterial infections certain bacteria are well known such as* S. aureus*,* E. coli*, and* Pseudomonas*. Recently most of bacterial strains become resistant to existing antibacterial compounds and pose major problem for treatment [16].* Candida albicans* is considered the most commonly occurring fungus responsible for infections [17]. In case of protozole infections leishmaniasis and malaria are main health problems around the world particularly in the developing countries [18].

Therefore, it is essential to screen various plants for their antimicrobial, antiprotozoal potential [14, 19]. Antimicrobial assays include agar well diffusion, disc diffusion, and broth dilution methods. Infections related to cancer also need attention due to emergence of resistance. Cytotoxicity and enzyme inhibitory studies can play an important role in drug development against cancer [20]. The brine shrimp lethality bioassay is important and effective assay to screen cytotoxic potential while various enzymes inhibition assays, for example, protein kinase inhibition, aromatase inhibition, and iNOS inhibition assays, are used for anticancer studies [21]. Protein kinases show a huge class of enzymes that play an important role in regulation of complex molecular mechanisms that control various functions of the cells, including survival, proliferation, and apoptosis. Many pathologic states appear due to abnormal protein kinase functions including cancer, inflammatory and autoimmune disorders, and cardiac diseases. Now the protein kinases are one of the major therapeutic targets [22]. For the treatment of cancer Protein kinase inhibitors are a well-known family of clinically effective drugs, especially in treating cancer [23].

The genus* Aloe* belonging to family Liliaceae exists almost all over the world and is extensively disseminated in the African and the eastern European continents. The genus* Aloe* has more than 400 species including globally traded* A. vera, Aloe ferox, *and* Aloe arborescens* [24]*. Aloe* genus is reported for many medicinal uses such as its use in treating constipation, gastrointestinal disorders, and immune system deficiencies.* Aloe vera *also showed pharmacological activities including antioxidant, antimicrobial, antitumor, hypoglycemic, hypolipidemic, and antidiabetic ones [25]. These properties are mainly contributed by inner gel of the leaves and presence of more than 200 different biologically active substances [24]. The medicinal and pharmacological potential of* A. vera *revealed that it is quite auspicious as a versatile therapeutic plant and should be further investigated. In this context, the present study was designed to evaluate total phenolics content,* in vitro *antioxidant and antimicrobial properties, cytotoxicity, and protein kinase inhibition activity in various fractions of* Aloe vera* leaf extract. Some of the well-known secondary metabolites of* A. vera* plant belonging to anthraquinone, resins, anthracenes, and polysaccharide class were used for in silico study against enzymes involved in hyphae formation of* Streptomyces*, hatching of* Artemia salina *larvae, and growth of leishmanial parasite.

## 2. Materials and Methods

### 2.1. Sampling

The plant samples were collected from Haripur, which lies between 34°-34′ and 34°–16′ North latitude and 77°-33′ and 73°-21′ East longitude, Khyber Pakhtunkhwa, Pakistan, September 2014, and is identified by taxonomist at Quaid-i-Azam University Islamabad. Plant samples were collected from private land by the verbal permission of the land owner to conduct this study. About 13 kg, fresh leaves were cleaned with tap water followed by distilled water and then chopped in the grinder and shade-dried with continuous agitation for 2 weeks.

### 2.2. Extraction

Approximately, 388g dried plant material was macerated in 16 liters of methanol and kept for ten days at room temperature with continuous stirring and shaking at least 4 to 5 times daily. The primary separation was done with muslin cloth followed by filtration using Whatman filter paper. Filtrate was dried and evaporated under reduced pressure at 40°C in a rotary evaporator, and crude extract (20g) was stored for further analysis.

### 2.3. Fractionation by Column Chromatography

The crude extract was fractionated by using column chromatography [26]. After TLC optimization methanol and chloroform were selected for column chromatography. Concentrated solution of crude extract was prepared in selected solvents and loaded on blank silica gel (1g x 1.5g) to prepare sample for column chromatography.

Silica gel (60, 70-230 mesh, MERCK) was used as stationary phase and the variation of solvent combinations of increasing polarity was used as mobile phase. The bottom of the glass column was stocked with cotton pad and slurry (600 g of silica gel and 600 mL of chloroform) was carefully poured into the column with continuous tapping. Then previously prepared dry power of extract and silica gel was poured on the top of the column and column packing was completed by putting blank silica on the top. Solvent was poured from the top and opened the tap of the column. Elution of the extract was done with 400 mL of each solvent combination (methanol : Chloroform), that is, 0:100, 2:98, 3:97, 5:95, 7:93, 10:90, 13:87, 16:84, 19:81, 23:77, 29:71, 35:65, 40:60, 50:50, 60:40, and 70:30 v/v. In total 55 elution fractions were collected in 100 mL glass bottles. The fractions present in each bottle were studied by TLC method to mix the closely related fractions on the basis of spots observed in UV light. Fractions that showed the same spots were pooled together and total ten fractions were obtained, that is, AV1, AV2, AV3, AV4, AV5, AV6, AV7, AV8, AV9, and AV10.

### 2.4. Estimation of Total Phenolics

Total phenolics content (TPC) in different fractions of* A. vera* leaf (AV1-AV10) was determined following the method of [27] with slight modification. In brief, 20 *μ*L of each fraction was taken in 96-well plate followed by addition of 90 *μ*L diluted Folin-Ciocalteu reagent in each well. The mixture was incubated for 5 min at room temperature and 90 *μ*L of 6% sodium carbonate solution was added and again incubated for 1 hour at room temperature. The absorbance was measured at 630 nm wavelength on microplate reader. Final value of total phenolics content was expressed as Gallic acid equivalent (GAE) and data were expressed as ± SD for triplicate analysis.

### 2.5. Estimation of Total Flavonoids

Total flavonoids content (TFC) was estimated by ammonium chloride calorimetric method as described by [28]. Briefly, 20 *μ*L of each fraction in triplicate was taken in 96-well plate followed by addition of 10 *μ*L of potassium acetate. Then, 10 *μ*L of aluminum chloride (10%) was added to the reaction mixture and constituted up to 200 *μ*L using distilled water. Then, mixture was incubated for 30 minutes at 25°C. Quercetin was used as positive control and DMSO was used as negative control or blank. Absorbance was measured at 405 nm on microplate reader. The TFC value was expressed as Quercetin equivalent and data were presented as ± SD for triplicate analysis.

### 2.6. Assessment of Antioxidant Activity

Antioxidant activity in different fractions of* A. vera *leaf was determined using different assays, such as 2, 2-diphenyl-1-picrylhydrazyl (DPPH), ferric ion reducing power assay, and phosphomolybdenum complex assay.

#### 2.6.1. DPPH Assay

The DPPH free radical scavenging assay is established to evaluate the scavenging potentials of antioxidants against the stable 2, 2-diphenyl-1-picrylhydrazyl (DPPH) radical. For the estimation of antioxidant, scavenging activity, DPPH assay is thought to be an authentic and simple assay [29]. The DPPH free radical scavenging activity of* A. vera *leaf fractions was measured by following the method of [27]. Briefly, 10 *μ*L of the analyte solution was added in 96-well plates, followed by addition of 190 *μ*L of DPPH testing agent. Ascorbic acid (1mg/ml) was positive control while DMSO was used as negative control. Absorbance was measured at 517 nm on microplate reader after one hour of incubation (37°C). The percentage DPPH radical scavenging activity was calculated using the formula

(1) $$\begin{array}{c} \%\;DPPH\;scavenging=\left(\;1-\frac{As}{Ac}\;\right)*100. \end{array}$$

where As is the absorbance of sample and Ac is the absorbance of control. Data were expressed as ± SD for triplicate analysis.

#### 2.6.2. Ferric Ion Reducing Power Assay

Reducing power is powerful impression of antioxidant activity of a compound [30]. Compounds that have to reduce may interact with potassium ferricyanide (Fe3+) and reduce it to potassium ferrocyanide (Fe2+), which subsequently interacts with ferrous chloride in order to generate complex of ferric ferrous which at 700nm wavelength shows maximum absorption [31]. The ferric ion reducing power was estimated by following the method as described earlier [32]. Briefly, 200 *μ*L of each fraction was taken in Eppendorf tube, followed by the addition of 400 *μ*L of 0.2M Phosphate buffer (pH 6.6) and 500 *μ*L of 1% Potassium ferricyanide. Then mixture was incubated for 20 min at 50°C. Then 400 *μ*L of 10% Trichloroacetic acid solution was mixed and centrifuged at 3000 rpm for 10 min. Then 150 *μ*L of supernatant was added in 96-well plates. After that 50 *μ*L of 0.1% of ferric chloride was mixed to each well containing sample mixture. The 200 *μ*L of ascorbic acid (1 mg/mL) was used as positive control and equal volume of DMSO was used as negative control. The absorbance was taken at 630 nm on microplate reader and results of ferric ion reducing power were presented as AA equivalent *μ*g/mL. Data were expressed as ± SD for triplicate analysis.

#### 2.6.3. Phosphomolybdenum Complex Assay

The total antioxidant potential is also accessed through generation of complex compound called phosphomolybdenum. In this assay the Mo (VI) is reduced to Mo (V) through the test sample and further generation of Mo(V) green phosphate complex at a pH which is acidic [33]. Total antioxidant potential of AV1-AV10 fractions was determined by phosphomolybdenum complex assay as explained previously [32]. In short, 100 *μ*L of each fraction was taken in 96-well plate and 900 *μ*L of reagent solution [4 mM ammonium molybdate, 28 mM sodium phosphate, 0.6 M sulfuric acid, 1.63 mL conc. sulfuric acid, 1.6795g NaH_2_SO_4_, and 0.247g ammonium molybdate and 50 mL distilled water] was added. The test blend was incubated at 95°C for 90 min. Ascorbic acid (1 mg/mL) solution was positive control, while DMSO was used as negative control. The reading was taken at 630 nm wavelength on microplate reader and results of total antioxidant activity were presented as AA equivalent *μ*g/mL. Data were expressed as ± SD for triplicate analysis.

### 2.7. Antibacterial Assay

Antibacterial activity of* A. vera *leaf fractions against gram positive* Staphylococcus aureus* (ATCC 6538) and* Micrococcus luteus *(ATCC 10240) and gram negative* Escherichia coli* (ATCC 15224) and* Pseudomonas aeruginosa* was determined by microtiter plate method [34]. The bacterial culture was formulated in nutrient broth and incubated for 12 hrs and then stored in refrigerator at 4°C. Before testing, culture was transferred to incubator for 14 hrs and diluted 10 folds with nutrient broth (1:10). 5 *μ*L of the sample solution (4 mg/mL sample solution + 20% DMSO and nutrient broth) was taken in 96-well plate. Then 195 *μ*L of freshly diluted inoculums were added to each well and the final test concentration was 100*μ*g/mL. The active sample was tested at lower concentration by 3 times' serial dilution, that is, 33.3, 11.1, and 3.7*μ*g/mL. The Cefixime monohydrate was used as positive control at 10, 3.33, 1.11, and 0.37 *μ*g/mL, while 5*μ*L of 20% DMSO in nutrient broth was taken as negative control. Absorbance was measured at 630 nm after 24 hrs incubation through microplate reader and percentage inhibition was calculated.

### 2.8. Antifungal Assay

Four fungal strains,* Mucor *species,* Aspergillus flavus, Fusarium solani*, and* Aspergillus niger*, were used to test the antifungal potential in studied samples using disc diffusion as explained earlier [35]. The fungal strains were stored on SDA at 4°C. Terbinafine (4mg/mL in DMSO) and pure DMSO were used as positive and negative control, respectively. Terbinafine disc concentration was 20 *μ*g. Four growing of fungal strains were cultured in Sabouraud dextrose agar (SDA) having 6.5 pH. 5 *μ*L of each crude extract solution was spew on the surface of filter paper discs and were implanted on the surface of the media in the Petri plate. The concentration of the sample and control was 100*μ*g/disc. The prepared Petri dishes were incubated for 72 hours at 28°C and after incubation the inhibition zones were measured.

### 2.9. Antileishmanial Activity

The in vitro antileishmanial activity of* Aloe* fractions was determined by using the method as described before [36] with slight adjustments. The leishmanial strain (*Leishmania tropica *KWH23) was cultured in medium 199 containing 10% heat-inactivated Fetal Bovine Serum maintained at 24 ± 1°C for 6-7 days. The amphotericin B was used as positive control drug and DMSO was used as negative control. In 96-well plates the stock solutions (4 mg of crude fraction + 1000 *μ*L of DMSO) were diluted serially. The DMSO was used as negative control and Amphotericin B was used as positive control. The 96-well plate was incubated for 72 hours at 24°C. After the incubation, 15 *μ*L of assay culture was transferred to counting chamber (Neubauer) for live promastigotes counting using light microscope. Table curve 2D version 4 was used for LC_50_ calculation. Data were expressed as ± SD for triplicate analysis.

### 2.10. Cytotoxicity Assay

The cytotoxic potential in* A. vera leaf *fractions was estimated through brine shrimp lethality test as discussed earlier [37] with some modifications. At first, stock solution of samples was formulated (100mg crude fraction/mL DMSO); then 500, 250, 100, and 10 *μ*g/mL dilutions were formulated. Different dilutions of Doxorubicin (1mg of doxorubicin + 1 mL DMSO), that is, 10, 1, and 0.1 *μ*g/mL, were used as standard drug. A shallow rectangular tray (22x32 cm) already was filled with simulated sea water (38g sea salt/L of distilled water) in which brine shrimp eggs (*Artemia salina *L. purchased from Sara, Heidelberg, Germany) hatched. The tray was consisting of two portions, one of which was large and the other was small. Each portion was separated from the other through a wall consisting of many holes. In the large portion of the tray brine shrimp's eggs were spread on the surface of simulated sea water. After spreading the brine shrimp's eggs, the large portion of the tray was concealed with aluminum foil. The surface of smaller portion was lighted up with a lamp and hatching started after 24-26 hours. The newly brine shrimp larvae (nauplii) traveled towards the light portion. These nauplii were taken from the tank and shifted to the beaker by Pasteur pipette.

50 *μ*L of sample solution with four concentrations (500, 250, 100, and 10*μ*g) was poured into respective well of 96-well plate, followed by addition of 200 *μ*L of the simulated sea water and mixed carefully. Ten shrimps were counted under a 3x magnifying glass and then transferred to each well with the help of Pasteur pipette and 300 *μ*L volume was maintained for each well to accomplish the required concentration of crude sample. The microplates containing shrimps were incubated at room temperature for 24 hrs. Then shrimps were taken out from the well with Pasteur pipette and survivor rate was counted under a 3x magnifying glass with 3x resolution against a background that is lighted. The values of LD_50_ (sample concentration required to kill 50% of shrimps) were calculated by using Table curve 2D version 4.

### 2.11. Protein Kinase Inhibition Assay

The protein kinase inhibition potential was studied through a method established on the restriction of hyphae formulation in* Streptomyces* as explained before [38, 39]. The sample solution was formulated by mixing 4 mg fractions/1000*μ*L DMSO. In 20 mL of tryptic soya, broth culture was grown for 3 to 4 days with shaking at 30°C. The production of hyphae inhibition test on* Streptomyces* was carried out. The* Streptomyces* mycelia fragments were dispersed on the surface of ISP4 agar media plates. Then 5 *μ*L of the test sample was applied to each disk and placed carefully on the agar surface previously seeded with* Streptomyces*. Two types of zones were appeared, that is, clear and bald type around the area of paper disk, which were measured after 30 to 48 hrs of incubation. The inhibition zone greater than 9 mm revealed that the sample is active. The Surfactin and DMSO were used as positive control and negative control, respectively.

### 2.12. Molecular Docking

Chemical structures of compounds 1-10 as mentioned in Table 1 were selected from NCBI database and drawn by ChemBioDraw. The legends were converted into MOL format for further use. The 3D crystal structure of serine protease (UniProt ID: A8D853) (PDB ID: 2HLC), trypanothione reductase (PDB ID: 2W0H), and tyrosinase (PDB ID: 3NM8) enzymes involved in hatching of* Artemia* larvae, growth of Leishmania, and formation of Streptomyces, respectively, was accessed and downloaded from Protein Data Bank (PDB) database [http://www.rcsb.org/pdb/home/home.do]. The active sites of target proteins were analyzed using the Molecular Operating Environment (MOE) software [https://www.chemcomp.com/MOE-Molecular_Operating_Environment.htm]. An active site was defined from the coordinates of the ligand in the original target protein sites.

A computational ligand-target docking approach was used to determine structural complexes of receptor targets with ligands molecules in order to understand the structural basis of these proteins targets specificity. Finally, docking was carried out by Molecular Operating Environment (MOE) software. The energy of interaction of these compounds with the protein targets is assigned “grid point” [39].

## 3. Statistical Analysis

All tests relevant to composition and properties assessment of* A. vera *were performed in triplicate. Data were presented as mean ± SD from at least three replicates.

## 4. Results and Discussion

### 4.1. Total Phenolics and Flavonoids Contents

The measured values of TPC and TFC in different fractions of* A. vera *(AV1 to AV10) are given in Table 2. AV4 fraction exhibited the highest level of total phenolics content (332.4 ± 32.6 *μ*g GAE/mg), followed by AV5, AV3, and AV6 (304.6 ± 29.6, 180.3 ± 21.9, and 157.8 ± 15.4 *μ*g GAE/mg, resp.). The lowest value of total phenolics content was showed by AV10 (97.95 ± 21.5 *μ*g GAE eq./mg). These findings are in agreement with each other as reported previously [40, 41]. It has been reported that plants with high phenolic contents have wound healing ability and reduce inflammation. However, method of extraction has a great impact on the value of total phenolic content, so more phenolics were determined in ethanol and chloroform extracts compared to water extract [42, 43].

The anti-inflammatory and antimicrobial potential of plants is attributed to flavonoids content. In the studied fractions, TFC content ranged from 18.46 ± 7.68 to 87.54 ± 15.5*μ*g QE/mg. AV3 fraction exhibited the highest TFC, whereas the lowest value was calculated for AV7.In* A. vera *fractions, measured levels of TFC were comparatively lower than those reported previously [42]. Furthermore, type of solvent may also contribute in the extract amount so more TFC content was reported in methanol-chloroform extract than aqueous extract [35, 41].

### 4.2. Antioxidant Potential

**Figure 1:**
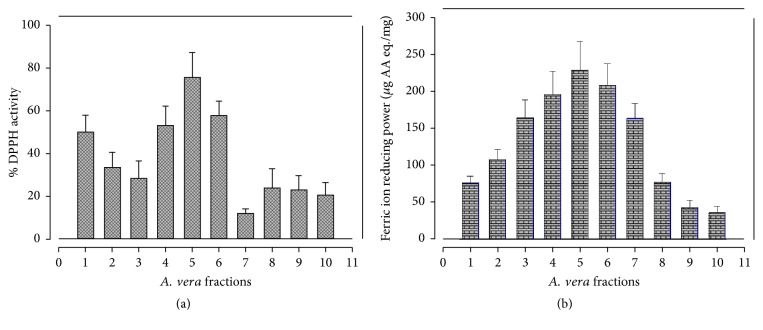
Free radicals scavenging potential in different fractions of* A. vera *leaf. (a) DPPH activity. (b) Ferric ion reducing antioxidant potential.

DPPH radical scavenging, ferric ion reducing potential and total antioxidant activity assays were conducted to determine the antioxidant potential in methanolic fractions of* A. vera *leaf. The percentage of DPPH radical scavenging capacity of various fractions as shown in Figure 1(a) ranged from 11.82 to 75.54%. AV5 fraction has the highest DPPH scavenging activity (75.54 ± 11.6%), followed by AV6 and AV4 (57.87 ± 6.53% and 52.99 ± 9.01%, resp.). These values were comparable to previously reported levels in the ethanolic extract of* A. vera *leaf [42]; however, they were a little lower than those reported earlier [41]. However, more DPPH radical scavenging activity has been reported in the methanolic and ethanolic extracts of* A. vera* leaf and pulp than n-hexane and aqueous extracts [44, 45].

**Figure 2:**
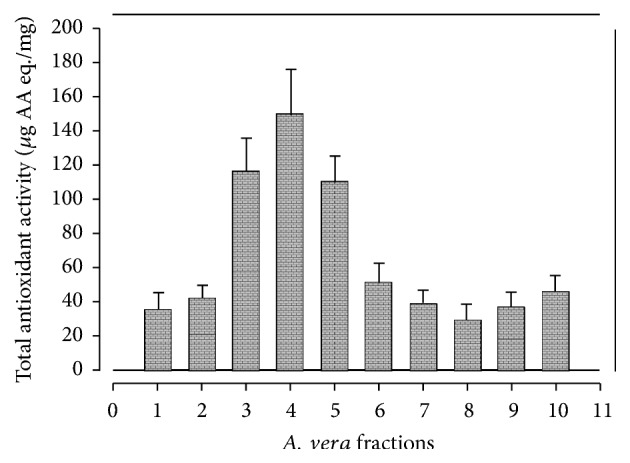
Total antioxidant activity of* A. vera *leaf fractions.

Results of ferric ion reducing antioxidant power (FRAP) are shown in Figure 1(b). All tested fractions showed significant ferric ion reducing ability; however AV5 fractions exhibited the highest potential 228.9 ± 39.1*μ*g AA eq./mg to reduce ferric ion, followed by AV6 (208.6 ± 29.3 *μ*g AA eq./mg) and AV4 (195.9 ± 31.2 *μ*g AA eq./mg). However, AV10 fraction exhibited the lowest FRAP value 36.18 ± 7.96*μ*g AA eq./mg. In a study on leaf skin of* A. vera* ethanol-chloroform extract depicted the highest reducing power activity compared to ethyl acetate, butanol, and n-hexane extract [46]. Therefore, variation in FRAP values might be due to difference in plant parts used, harvesting time, geoclimatic conditions, and solvent system used for the extraction as reported [47]. The results of total antioxidant capacity (TAC) determined by phosphomolybdenum complex assay are given in Figure 2. The total antioxidant activity in AV1 to AV10* A. vera *leaf fractions ranged from 28.77 ± 9.36 to 150.4 ± 25.8*μ*g AA eq./mg, with the highest TAC value in AV4 and the lowest in AV8 fraction. Three fractions AV4, AV3, and AV5 exhibited the highest TAC. These findings are in compliance to [46].

### 4.3. Antimicrobial Potential

The antibacterial potential of studied samples given in Table 3 revealed that crude fractions were not much effective against* E. coli, *but relatively good results were obtained for* P. aeruginosa*. The most significant inhibition was shown by AV8, AV6, AV10, and AV9 fractions against* P. aeruginosa *with MIC values of 0.70, 0.71, 7.72, and 8.68*μ*g/mL, respectively. The AV4, AV7, and AV9 fractions exhibited good response against* M. luteus *with MIC values of 103, 161, and 250*μ*g/mL, respectively. In the previous studies regarding* A. vera* DMSO gel extract, ethanolic extract was found active against* E. coli*,* P. aeruginosa*, and* S. aureus *[48–50]. However, antibacterial potential of* A. vera *fractions in the present evaluation against* E. coli* was not in support with reported data. However, Lawrence et al. [50] reported significant potential in methanolic extract of* A. vera *against* E. coli *which is partially in agreement with the current study.

The antifungal activity of* A. vera *fractions against four strains, that is,* Aspergillus niger, Mucor* specie,* Aspergillus flavus*, and* Fusarium solani *is mentioned in Table 4. All the tested fractions exhibited minor effects against fungal strains which is not in complete agreement with previous reports [51, 52]. This variation of biological potential may be due to solvent use, harvesting time, seasonal variation, and ecological factors.

**Figure 3:**
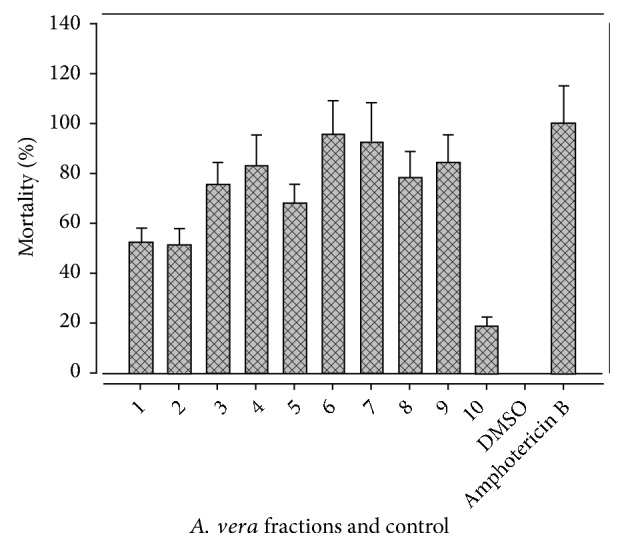
Antileishmanial activity of* A. vera *leaf fractions.

The antileishmanial activity carried out at 50 *μ*g/mL concentration in different fractions of* A. vera *leaf extract and results are given in Figure 3. The highest value of mortality was shown by AV6 fraction (95%), followed by AV7, AV9, and AV4 (92, 84, and 83%, resp.), whereas the lowest value was shown by AV10 (18%). The results were comparable with previous studies [53–55].

### 4.4. Cytotoxicity of* A. vera* Fractions

The brine shrimp lethality assay is considered as the most appropriate assay for the pharmacological active crude plant extract which may exhibit lethality against newly hatched nauplii [56]. In the present study, cytotoxicity of* A. vera *leaf fractions was determined by Brine Shrimp Lethality assay and results in LD_50_ (*μ*g/mL) value are mentioned in Table 5. The fractions with low LD_50_ value are more active and have high cytotoxic potential. Therefore, AV6 fraction depicted the highest cytotoxicity with LD_50_ value of 0.530*μ*g/mL, followed by AV4, AV5, and AV3 (4.710, 4.710, and 9.070 *μ*g/mL, resp.). A study carried out on cytotoxicity of* A. vera* was reported against brine shrimp and LD_50_ was estimated over 500*μ*g/mL [20], which is partially correlated with the current study. However, brine shrimp lethality activity in methanolic extract of* Melia azedarach *stem bark with3.27*μ*g/mL LD_50_ value [57] is in agreement with our findings.

### 4.5. Protein Kinase Inhibitions

**Figure 4:**
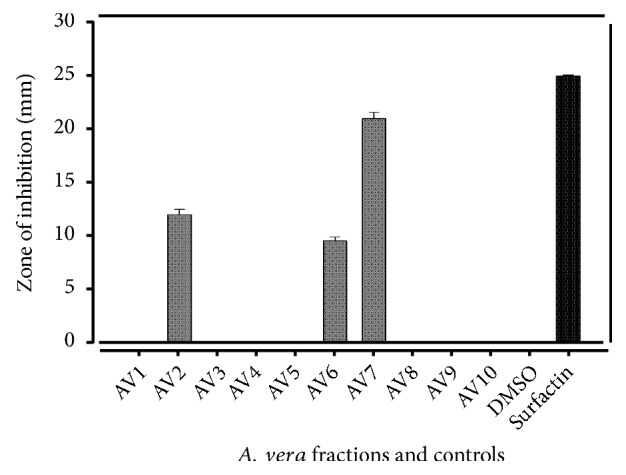
Protein Kinase inhibitions in* A. vera *leaf fraction.

Figure 4 depicted protein kinase inhibition (PKI) activity in the methanolic fractions of* A. vera *leaf. At 4mg/mL concentration, only three fractions AV7, AV6, and AV2 showed PKI activity. The AV7 fraction exhibited the highest zone of inhibition (21 ± 0.58mm), followed by AV2 and AV6 (12 ± 0.45 and 9.5 ± 0.37mm, resp.). The results are strongly supported by a study of Yao and coworkers, which was carried out on evaluation of hyphae formation inhibition in* Streptomyces *85E, and isolated compounds showed 21mm zone of inhibition at 80*μ*g/disk and it was hypothesized that the compounds prevent the formation of hyphae in* Streptomyces* 85E, which may inhibit cancer proliferation [38].

Comparatively, measured levels of secondary metabolites and biological activities particularly antioxidant potential in different fractions of* A. vera *were less than those in previous reports. Such variations may be attributed to diverse factors including genetic variation in* A. vera *reported from various regions, harvesting time and storage conditions, difference in geoclimatic conditions, and growing environment such as temperature, precipitation, humidity, altitude, salinity, and drought stress along with the analytical techniques used.

### 4.6. Molecular Docking Study

#### 4.6.1. Isolation of Binding Sites

**Figure 5:**
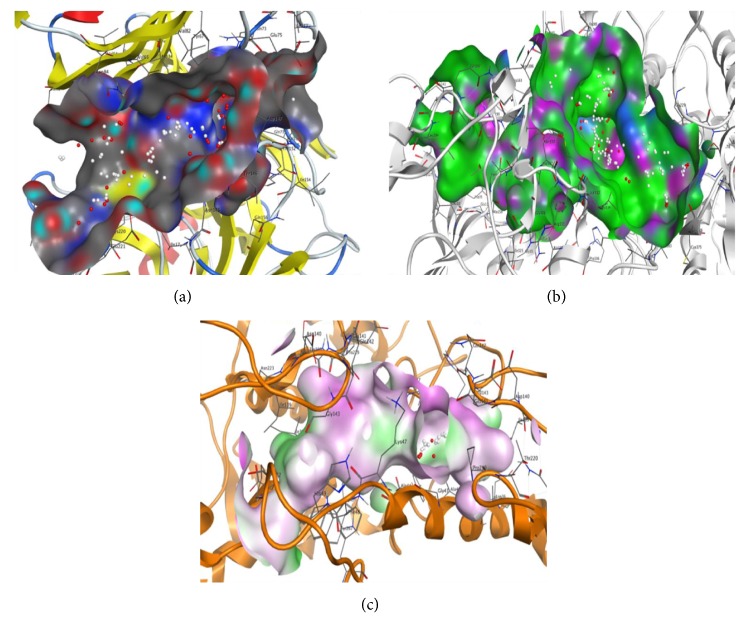
Potential isolated binding sites of each target protein analyzed by Molecular operator Environment (MOE) software. (a) Serine protease. (b) Trypanothione reductase. (c) Tyrosinase.

Potential isolated binding sites of each target protein were analyzed by Molecular operator Environment (MOE) software. The possible binding sites residues of serine protease (UniProt ID: A8D853) (PDB ID: 2HLC) were as follows: ILE17, TRP41, GLN73, TYR74, TRP141, GLY142, GLN143, SER144, ASN145, THR146, ASP147, THR152, VAL153, ILE154, GLN156, CYS191, PHE192, GLY193, GLY217A, ALA218, GLY219, CYS220, GLU221, and SER222 (Figure 5(a)). Similarly in case of trypanothione reductase (PDB ID: 2W0H), binding site residues were LEU10, GLY11, ALA12, GLY13, SER14, GLY15, GLY16, VAL34, ASP35, VAL34, ASP35, VAL36, ALA46, ALA47, GLY49, GLY50, THR51, CYS52, VAL55, GLY56, CYS57, LYS60, GLY125, PHE126, GLY127, GLU141, ALA159, THR160, GLY161, SER162, TRP165, ALA200, GLU202, PHE203, GLY229, PHE230, ASP231, LEU283, ALA284, ILE285, GLY286, ARG287, VAL288, PRO289, ARG290, SER291, GLN292, GLN292, ALA293, LEU294, ASN306, ILE325, GLY326, ASP327, VAL328, ASN330, ARG331, VAL332, MET233, LEU334, THR335, PRO336, and ALA338 (Figure 5(b)). Binding site residues of tyrosinase (PDB ID: 3NM8) were ASP36, ILE39, ALA40, TRP41, GLY43, ALA44, LYS47, PHE48, HIS49, ILE139, ASP140, GLU141, GLN142, GLY143, PRO219, THR220, ASN223, and TYR267 (Figure 5(c)). Protein geometric arrangement of amino acid residues in allowed and disallowed regions indicates the quality of target proteins.

#### 4.6.2. Docking Analysis

**Figure 6:**
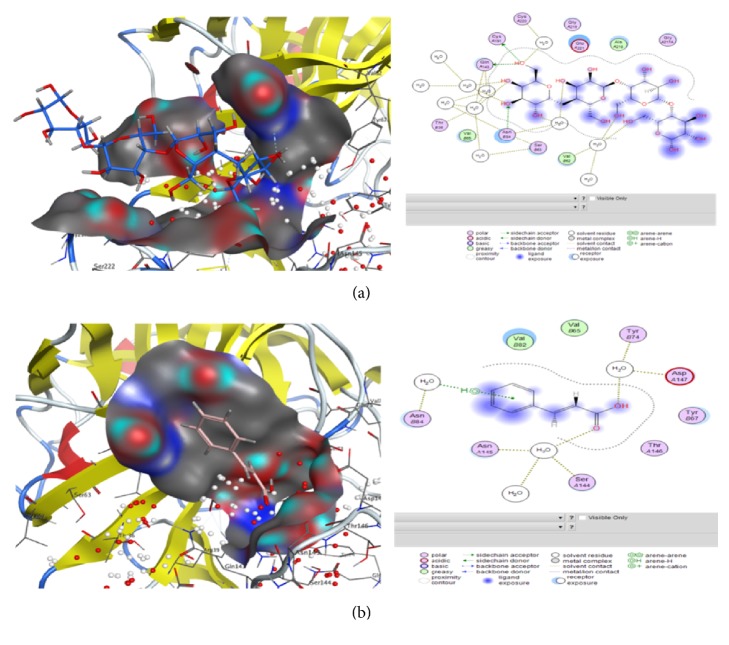
Views of molecular docking and information of ligands interaction with atoms of serine protease target performed by Molecular Operating Environment (MOE) software comp-4 (a) and comp-9 (b) as mentioned in Table 1.

**Figure 7:**
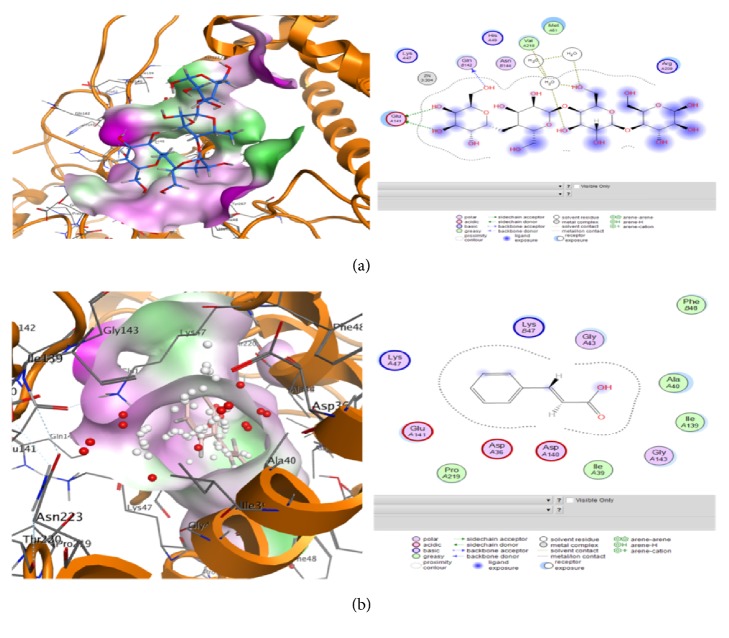
Views of molecular docking and information of ligands interaction with atoms of tyrosinase target performed by Molecular Operating Environment (MOE) software comp-3 (a) and comp-8 (b) as mentioned in Table 2.

Molecular docking was performed to find ideal ligand orientation with protein for stable complex formation. Strength of association between ligand and protein is evaluated on the basis of scoring function and minimum binding energy. Computational ligand-target binding approach was used in analyzing structural complexes of compounds with three selected enzymes in order to interpret structural basis of target protein specificity. The interaction energy of compounds with target enzyme is assigned, “grid point.” Finally, these ligands were docked with the potential active sites of target molecules. Compounds 3 (mannan), 4 (isoaloresin D), and 9 (cinnamic acid) showed good results against serine protease with binding energy above -5.0 (Table 6/Figures 6(a) and 6(b)) while rest of the compounds also showed binding potential. These finding are also correlated with the fact that* A. vera* extract showed cytotoxic activities against cell lines [58]. In case of effects of these compounds when analyzed against trypanothione reductase enzyme responsible for leishmanial parasite's growth it was observed that compounds 3 (mannan), 5 (emodin), and 6 (aloe barbendol) showed the lowest binding energy (Table 7) and against tyrosinase compound 8 (aloetic acid) showed better results while compounds 2 (aloe emodin), 3(mannan), 4 (isoaloresin D), and 9 (cinnamic acid) also showed some activity indicating their potential for Streptomyces inhibition (Table 8/Figures 7(a) and 7(b)). These findings are correlated with previous reports that* Aloe vera* plant contains anthraquinones, anthrones, chromones, and polysaccharides responsible for anticancer, antileishmanial, antibacterial, and antioxidant potential of plant [53, 59]. Docking results indicating the lowest binding energy cluster were considered as representative binding states. The minimum binding energies showed that target proteins were docked successfully with ligand molecules.

## 5. Conclusion

The present study was focused to assess* in vitro* protein kinase inhibition, brine shrimp lethality assay, and antioxidant activity of different factions (AV1 to AV10) of* A. vera* leaf extract. The AV4, AV5, AV6, and AV7 methanolic fractions depicted significant TPC and TFC contents and antioxidant and antimicrobial activities along with cytotoxicity and protein kinase inhibition. The Protein Kinase Inhibitory assay was performed first time and AV7 depicted the highest value of 21 ± 0.50mm. Our findings revealed that* A. vera* is a versatile medicinal plant with significant biological activities and could be used for future drug against cancer and leishmaniasis.

## Figures and Tables

**Table 1:** Details of compounds used for in silico analysis.

S #	Code	Name of compound
1	Compound 1	Aloin (anthraquinone glycoside)
2	Compound 2	Aloe emodin (anthraquinone)
3	Compound 3	Mannan (polysaccharide)
4	Compound 4	Isoaloresin D (chromones)
5	Compound 5	Emodin (hydroxyl anthraquinone)
6	Compound 6	Aloe barbendol
7	Compound 7	Physcion (anthraquinone derivative)
8	Compound 8	Aloetic acid
9	Compound 9	Cinnamic acid
10	Compound 10	Galactan

**Table 2:** TPC^*∗*^ and TFC^*∗*^ contents in different fractions of *A. vera*.

Fractions	TPC (*μ*g GAE. eq/mg)	TFC (*μ*g QE. eq/mg)
AV1	139.7 ± 15.1	51.87 ± 12.0
AV2	136.2 ± 13.3	51.28 ± 7.89
AV3	180.3 ± 21.9	87.54 ± 15.5
AV4	332.4 ± 32.6	60.47 ± 13.7
AV5	304.6 ± 29.6	51.03 ± 9.87
AV6	157.8 ± 15.4	32.47 ± 6.57
AV7	119.7 ± 13.3	18.46 ± 7.68
AV8	104.4 ± 12.0	19.49 ± 5.79
AV9	113.3 ± 19.5	22.67 ± 4.60
AV10	97.95 ± 21.5	24.87 ± 3.12

TPC:^*∗*^ total phenolics; TFC:^*∗*^ total flavonoids.

**Table 3:** Antibacterial activity of *A. vera *leaf's fractions.

Fractions	Minimum inhibitory concentration (MIC)							
	*M. luteus*		*S. aureus*		*E. coli*		*P. aeruginosa*	
	% inhibition	MIC (*μ*g/mL)	% inhibition	MIC (*μ*g/mL)	% inhibition	MIC (*μ*g/mL)	% inhibition	MIC (*μ*g/mL)
AV1	57.93	325	69.15	250	55.95	673	76.00	60.20
AV2	49.61	1000	60.26	500	57.02	874	70.26	18.60
AV3	63.14	500	73.76	250	48.43	1000	80.25	10.02
AV4	63.40	103	67.44	<125	43.80	>1000	77.10	64.66
AV5	49.61	1000	66.75	<125	51.19	971	77.10	123.9
AV6	48.07	1000	69.15	<125	44.49	>1000	69.24	0.710
AV7	54.57	161	67.69	250	45.55	1000	70.42	125.0
AV8	48.78	1000	65.04	125	42.17	>1000	76.71	0.700
AV9	52.26	250	62.74	125	47.24	1000	74.82	8.670
AV10	53.35	1000	70.60	250	41.54	100	74.04	7.720
Cefixime	86.93	0.58	100.2	1.01	51.19	0.58	86.93	0.200

**Table 4:** Antifungal activity of *A. vera *leaf's fractions.

Fractions	Zone of inhibition (mm)			
	*A. niger*	*Mucor* spp.	*A. flavus*	*F. solani*
AV1	---	8.319 ± 1.2	7.198 ± 0.7	---
AV2	---	---	6.264 ± 1.3	7.245 ± 1.9
AV3	---	9.017± 1.5	8.451 ± 1.6	9.364 ± 2.0
AV4	---	10.25 ± 1.3	6.137 ± 0.7	8.170 ± 1.3
AV5	---	7.352 ± 0.9	9.326 ± 1.5	10.03 ± 0.6
AV6	---	8.036 ± 0.7	7.367 ± 0.8	9.217 ± 1.5
AV7	---	8.247 ± 1.3	- - -	8.612 ± 0.8
AV8	---	9.364 ± 1.7	8.353 ± 1.7	---
AV9	---	---	8.632 ± 2.0	8.219 ± 0.5
AV10	---	11.15 ± 2.0	---	---
-ve. control (DMSO)	---	---	---	---
+ve. control (Terbinafine )	22.15 ± 1.9	24.37 ± 2.5	27.47 ± 1.9	25.19 ± 2.7

**Table 5:** Cytotoxic activity of *Aloe vera *leaf fractions.

Fractions	Mortality (%)				
	500 *μ*g/mL	250 *μ*g/mL	100 *μ*g/mL	10 *μ*g/mL	LD_50_ (*μ*g/mL)
AV1	67	62	58	38	35.05
AV2	69	60	56	41	49.72
AV3	80	67	60	50	9.070
AV4	80	70	62	54	4.710
AV5	80	72	63	52	4.710
AV6	75	69	64	62	0.530
AV7	73	55	54	47	64.13
AV8	50	50	42	37	412.8
AV9	40	30	0	0	> 500
AV10	60	55	50	35	107.6
Doxorubicin	100	94	89	70	0.270

**Table 6:** Energy values obtained during docking analysis of compounds as ligand molecules serine protease (PDB ID: 2HLC) enzyme as target molecules.

S. no.	mol	rseq	mseq	S	Rmsd-refine	E-conf	E-place	E-score1	E-refine	E-score2
1	COMPD1_2HLC	1	1	-4.522	4.681	168.3	-110.5	-20.26	-12.83	-4.522
2	COMPD2_2HLC	1	2	-3.696	4.430	33.88	-126.1	-18.64	-9.617	-3.696
3	COMPD3_2HLC	1	3	-5.279	4.460	338.4	-126.0	-26.34	-13.28	-5.279
4	COMPD4_2HLC	1	4	-5.249	3.720	146.2	-121.2	-16.25	-9.643	-5.249
5	COMPD5_2HLC	1	5	-3.651	3.198	32.87	-47.26	-17.16	-6.049	-3.651
6	COMPD6_2HLC	1	6	-4.149	3.672	40.29	-97.50	-16.75	-11.55	-4.149
7	COMPD7_2HLC	1	7	-4.204	3.778	47.10	-105.1	-19.84	-11.38	-4.204
8	COMPD8_2HLC	1	8	-3.785	2.240	-17.62	-53.66	-11.26	-8.136	-3.785
9	COMPD9_2HLC	1	9	-5.034	4.061	281.6	-116.6	-22.48	-16.40	-5.034
10	COMPD10_2HLC	1	10	-3.932	3.282	22.61	-45.39	-18.20	-7.129	-3.932

**Table 7:** Energy values obtained during docking analysis of compounds as ligand molecules trypanothione reductase (PDB ID: 2W0H) enzyme as target molecules.

S. no.	mol	rseq	mseq	S	Rmsd-refine	E-conf	E-place	E-score1	E-refine	E-score2
1	COMPD1_2W0H	1	1	-5.950	6.927	154.3	-140.6	-15.74	-3.376	-5.950
2	COMPD2_2W0H	1	2	-5.943	4.344	33.24	-128.4	-15.06	-17.51	-5.943
3	COMPD3_2W0H	1	3	-7.136	3.591	369.9	-192.9	-17.05	13.33	-7.136
4	COMPD4_2W0H	1	4	-1.220	2.907	195.1	-149.7	-13.59	70.64	-1.220
5	COMPD5_2W0H	1	5	-6.221	2.175	39.06	-103.4	-13.25	-8.982	-6.221
6	COMPD6_2W0H	1	6	-6.025	3.343	38.80	-115.4	-15.22	-15.99	-6.025
7	COMPD7_2W0H	1	7	-5.581	2.251	62.32	-133.5	-14.86	2.153	-5.581
8	COMPD8_2W0H	1	8	-5.148	3.238	-16.71	-72.16	-9.94	-19.81	-5.148
9	COMPD9_2W0H	1	9	-2.591	3.356	343.8	-182.9	-15.86	51.47	-2.591
10	COMPD10_2W0H	1	10	-5.958	2.460	25.19	-111.8	-14.74	-10.42	-5.958

**Table 8:** Energy values obtained during docking analysis of compounds as ligand molecules tyrosinase (PDB ID: 3NM8) enzyme as target molecules.

S. no.	mol	rseq	mseq	S	Rmsd-refine	E-conf	E-place	E-score1	E-refine	E-score2
1	COMPD1_3NM8	1	1	7.123	2.412	318.7	-131.1	-19.68	126.5	7.123
2	COMPD2_3NM8	1	2	-4.240	3.577	49.18	-131.2	-15.90	13.81	-4.241
3	COMPD3_3NM8	1	3	-4.964	6.082	338.2	-153.2	-23.94	-14.51	-4.965
4	COMPD4_3NM8	1	4	-4.963	5.343	124.6	-85.28	-14.32	-15.57	-4.963
5	COMPD5_3NM8	1	5	-2.949	3.719	56.49	-96.54	-15.63	25.46	-2.949
6	COMPD6_3NM8	1	6	-2.244	1.677	56.00	-94.59	-16.35	36.25	-2.245
7	COMPD7_3NM8	1	7	1.947	2.255	160.5	-135.3	-18.75	82.87	1.948
8	COMPD8_3NM8	1	8	-5.016	1.550	-16.19	-63.69	-10.49	-10.49	-5.017
9	COMPD9_3NM8	1	9	-4.328	3.061	306.2	-138.3	-19.44	-9.012	-4.328
10	COMPD10_3NM8	1	10	-3.495	2.725	27.07	-98.46	-17.34	23.28	-3.495

## Data Availability

The data used to support the findings of this study are included within the article.
